# A low dose of naloxone mitigates autoimmune hepatitis by regulating TLR4/NF-κB and Nrf2/HO-1 signaling pathways

**DOI:** 10.1007/s10787-023-01327-5

**Published:** 2023-09-03

**Authors:** Kawther Magdy Ibrahim, Hebatalla I. Ahmed, Laila Ramadan, Amany Balah

**Affiliations:** 1 Department of Pharmacology & Toxicology, Faculty of Pharmacy, Egyptian Russian University, Cairo, Egypt; 2 Department of Pharmacology & Toxicology, Faculty of Pharmacy (Girls), Al-Azhar University, Cairo, Egypt

**Keywords:** Concanavalin A, Naloxone, Autoimmune hepatitis, TLR4, NF-κB, Nrf2/HO-1

## Abstract

Naloxone is a non-selective opiate receptor antagonist that is mainly used in the management of acute opioid overdose or intoxication. Previously, naloxone has been shown to have anti-inflammatory and antioxidant properties. Concanavalin A (Con A) model is a common and well established animal model of autoimmune hepatitis that closely resembles the pathological alterations that occur in human. The present study demonstrates that a low dose of naloxone (LD NX) has the ability to improve hepatic function and attenuate hepatic damage induced by Con A as indicated by a clear reduction in serum aminotransferase, bilirubin and enhancement of albumin production as well as liver pathological changes. Also, The proinflammatory cytokines, tumor necrosis factor-α (TNF-α), interferon- γ (IFN-γ), interleukin-6 (IL-6) and interleukin-1β (IL-1β) were highly suppressed in animals pretreated with LD NX via interference with TLR4/NF-κB as well as JNK signaling pathways. Furthermore, oxidative stress was highly attenuated in animals pretreated with LD NX as indicated by high reduction in hepatic MDA and an increase in Nrf2, HO-1 expression and subsequent production of the endogenous antioxidants, SOD, CAT and GSH. Collectively, this study demonstrates that LD NX has the ability to mitigate Con A-induced autoimmune hepatitis via modulation of inflammatory cytokines secretion and interference with reactive oxygen species generation.

## Introduction

Autoimmune hepatitis (AIH) is a kind of inflammatory liver disease (Vergani and Mieli-Vergani 2004) with high morbidity and mortality rate (Que et al. 2023). It is characterized by persistent hepatic parenchymal damage as well as hypergammaglobulinemia, and filtration of activated T lymphocytes in the liver (Li et al. 2012). It is caused by an immune response of unknown origin that can lead to liver cirrhosis, hepatocellular carcinoma (HCC), liver transplantation or death (Gomes et al. 2021). AIH prevalence worldwide is increasing, and several studies have reported a prevalence of AIH in Europe, the USA and Asia. (Katsumi and Ueno 2022). AIH normally recognized during late stage of the disease. Due to limited knowledge about the onset and course of disease and need for chronic immunosuppression with significant side-effects there is a requirement for a good treatment for AIH (Jaeckel et al. 2011). The overall goal of AIH treatment is to induce and maintain complete suppression of the inflammatory activity and to prevent disease progression to cirrhosis and liver decompensation (Sucher et al. 2019). Although the exact underlying cause of AIH is still unknown, an abundant of data suggests that a variety of inflammatory cytokines released by activated T cells contribute to the liver injury development (He et al. 2016).

Concanavalin A (ConA) is a plant lectin derived from the seeds of jack beans (Canavalia brasiliensis) (Elshal and Hazem 2022; Sharawy et al. 2021). Con A model is a typical and well established model of autoimmune hepatitis in animals, which closely mimics the pathological changes that occur in human (Ji et al. 2019). There are several signaling pathways involved in the pathophysiology of Con A-induced AIH. Toll-like receptors (TLRs) play a crucial role in maintain liver health, moreover its expression was detected in different types of hepatocytes (Zhai et al. 2016). It has been reported that TLR4 plays an important role in the pathophysiology of Con A-induced liver injury (Sahin et al. 2013). Con-A injection causes T cell activation, which results in the differentiation and production of several inflammatory mediators e.g., tumor necrosis factor-alpha (TNF-α), interleukins (ILs) (Mounieb et al. 2017).

Recent research has shown that NF-κB (Nuclear Factor-kappa B) plays a critical role in Con-A-induced inflammatory hepatotoxic effects. Con-A has been shown to activate NF-κB and translocate it to the nucleus, where it increases the production and release of downstream cytokines such as TNF-α (Elshal and Hazem 2022; Tiers et al. 1998), that leads to an abnormal immune response against hepatocytes (Li et al. 2021) and overproduction of various inflammatory mediators causing the so-called cytokine storm and release of transaminases into the blood circulation (Elshal and Hazem 2022). Also, TNF-α has the ability to activate JNK signaling pathway that may contribute to reactive oxygen species (ROS)-induced cell death (Elshal and Hazem 2022). Another pathogenic mechanism that occurs concurrently with the previous one is oxidative stress, which represent a well-known cause of various hepatic damages. Increased inflammation, as well as the suppression of antioxidative protective transcription factors such as nuclear factor erythroid 2-related factor 2 (Nrf2), worsen Con-A-induced hepatic oxidative damage (Mohamed et al. 2022). Recent studies have shown ROS as a significant element in Con A-induced acute liver damage (Yang et al. 2022; Zhuang et al. 2016). It has been demonstrated that Con A administration causes a significant reduction in Nrf2expression and its target cytoprotective protein heme oxygenase 1(HO-1) (Sharawy et al. 2021). As a result, medications that inhibit crosstalk between these signaling pathways TLR4/NF-κB/Nrf2 can provide protection against Con A induced AIH.

Naloxone hydrochloride, a synthetic N-allyl derivative of oxymorphone (Handal et al. 1983) is a short-acting non-selective opioid receptor antagonist (Glass et al. 1994; Van Dorp et al. 2007). It is used to counteract opioid-induced respiratory depression (Van Dorp et al. 2007). Many studies have focused on different aspects of the pharmacological activities of naloxone other than its indications. It has been shown that the antinociceptive effects of low doses of naloxone are remarkable under inflammatory conditions. It has been reported that intrathecal injection of ultra-low dose naloxone enhances the antinociceptive effect of morphine via inhibition of microglial activation, pro-inflammatory cytokines TNF-α, IL-1β and IL-6 in the spinal cord of pertussis toxin-treated rats (Lin et al. 2010). Naloxone has been demonstrated to exert neuroprotective effect. The neuroprotective activity appears to occur when microglia activation in the brain and spinal cord is suppressed (Younger et al. 2014). In addition, naloxone reduces the formation of reactive oxygen species and other potentially neuroexcitatory and neurotoxic substances by inhibiting microglia activation. (Younger et al. 2014). The anti-inflammatory effect of opioid antagonists may also extend to the periphery, as indicated by decreased TNF- α, IL-6 and other inflammatory molecules in peripheral macrophages (Liu et al. 2006). Previously, it has been reported that low dose naltrexone acts as an immunomodulating agent by binding directly on the opioid growth factor receptor (OGFr) within immune cells (Li et al. 2018). Furthermore, it was suggested that naltrexone exerts its effects on the body via at least two different receptor mechanisms. Microglia are considered as CNS resident macrophages that are activated by various triggers (Li et al. 2018). In addition to antagonizing mu-opioids and other opioid receptors, Low-dose naltrexone also inhibited non-opiate receptors such as TLR-4 in macrophages and microglia. (Cant et al. 2017; Li et al. 2018). TLR4 activation in microglia increases the synthesis and release of TNF- α, IL1β, interferon-β1 (IFNβ1) and other inflammatory agents (Dara et al. 2023). Opiate antagonists act as a TLR4 antagonist (Monnet et al. 2020; Dara et al. 2023). Low-dose naltrexone has been demonstrated to be highly effective against autoimmune illnesses including lupus and arthritis (Xu et al. 2020). The current standard of care for AIH patients includes non-specific immune dampening medicines such as corticosteroids in conjunction with azathioprine (Ballegeer and Libert 2016) and liver transplantation, but unfortunately, the availability of liver donors, immunological suppression, and high expenses restrict the potential of liver transplantation. As a result, the development of safe and effective medicines is critical. Drugs that reduce the manifestation of AIH may help to prevent AIH complications (Taubert et al. 2018). To our knowledge, no study has explored the impact of a low dose of naloxone (LD NX) on AIH. Therefore, it was interesting to investigate the potential modulatory effect of LD NX on Con A-induced AIH in rats.

## Material and methods

### Animals

Forty male Wistar albino rats (170–200 g) were obtained from Nile Company (Co.) for Pharmaceutical and Chemical Industries, Egypt. The animals were fed a standard diet pellets and water was supplied ad libitum. The rats were housed in the animal house of Faculty of Pharmacy, Al-Azhar University for one week before the experiment for acclimatization. The experimental protocol used in this study was approved by the Institutional Animal Ethics Committee. (Committee reference number: 289).

### Drugs and chemicals

Concanavalin A (con A) was purchased from Alfa Aesar, Erlenbachweg 2, Kandel, (Germany). Naloxone hydrochloride was purchased from abcam Biotechnology, Cambridge, (UK). Alanine amino transferase (ALT), aspartate amino transferase (AST), albumin, and bilirubin were purchased from Bio-Med diagnostics, (USA). Reduced glutathione (GSH), superoxide dismutase (SOD), catalase (CAT), and thiobarbituritic acid reactive substance (TBARS) assay kits were purchased from Bio-diagnostic, (Egypt). Rat interferon-γ (IFN-γ) enzyme-linked immunosorbent assay (ELISA) kit, rat TNF-α ELISA kit, rat IL-6 ELISA kit and rat IL-1β ELISA kit were purchased from MyBioSource, San Diego, California, (USA), Cusabio, Houston, (USA), R&D Systems, McKinley Place NE, Minneapolis, (USA) and MyBioSource, San Diego, California, (USA), respectively. An antibody raised against Nuclear factor kappa B (NF-κB p65), c-Jun N-terminal kinase (JNK), Nuclear factor erythroid 2 (Nrf2), Heme oxygenase-1 (HO-1), TLR4 and β-actin were purchased from Thermo Fisher Scientific, Waltham, (USA). Goat Anti-Rabbit HRP linked IgG and Goat Anti-Mouse HRP linked IgG were obtained from abcam Biotechnology, Cambridge, (UK).

### Experimental design

Forty male albino rats were randomly allocated into four groups (10 rats/group) and assigned as follows: Group (I): Served as control where animals received intravenous normal saline (0.9%) via the tail vein. Group (II): The animals were injected with Con A via the tail vein (12 mg/kg) (Mounieb et al. 2017). Group (III): Rats received LD NX (5 μg/kg/daily, i.p.) (Tsuruoka et al. 1997) for 10 days before and concurrently during Con A administration. Group (IV): Rats were administered LD NX alone. Twenty-four hours after Con A injection, blood samples were collected from the retro-orbital plexus. Serum was separated by centrifugation at 1000 g for 10 min and used for the assessment of serum levels of ALT, AST, albumin, and bilirubin. After terminal bleeding, rats were sacrificed, and liver tissues were dissected and washed with ice-cold phosphate-buffered saline (PBS), and kept at −20 °C till used. Also, specimens from the three major lobes of each liver from the different treatment groups were fixed in formalin 10% for histopathological examination and immunohistochemical analysis.

### Liver function test

#### Assessment of serum ALT and AST levels

The levels of ALT and AST in serum were determined by commercial kits according to the manufacturer’s instructions (Bio-Med diagnostics company, USA).

#### Assessment of albumin and total bilirubin levels

Albumin and total bilirubin levels in serum were detected by commercial kits according to the manufacturer’s instructions (Bio-Med diagnostics company, USA).

#### Assessment of oxidative stress markers

##### Determination of lipid peroxides

The level of Malondialdehyde (MDA) in liver tissues was measured using a TBARS assay kit according to the manufacturer’s instructions (Bio-diagnostic, Egypt) as previously described (Mounieb et al. 2017).

#### Determination of endogenous antioxidants

##### Determination of SOD activity

Superoxide dismutase activity in hepatic tissues was measured by assay kit according to the manufacturer’s instructions (Bio-diagnostic, Egypt).

##### Determination of CAT activity

Catalase activity in hepatic tissues was measured by assay kit according to the manufacturer’s instructions (Bio-diagnostic, Egypt).

##### Determination of GSH content

The liver content of GSH was determined by assay kit according to the manufacturer’s instructions (Bio-diagnostic, Egypt).

### Assessment of inflammatory markers

The liver content of the inflammatory markers IFN-γ, TNF-α, IL-6 and IL-1β were detected by ELISA according to the manufacturer’s instructions (MyBioSource, San Diego, USA), (Cusabio, Houston, USA), (R&D Systems, McKinley Place NE, Minneapolis, USA) and (MyBioSource, San Diego, USA), respectively.

### Western blot analysis

For detection of NF-κB, JNK, Nrf2, HO-1 and β-actin, whole-cell lysates were prepared. Total cell extracts containing 50–100 µg of protein were prepared in sodium dodecyl sulfate (SDS) sample buffer. These extracts were then subjected to SDS–polyacrylamide gel electrophoresis (PAGE) and Western blot analysis was performed. In general, each sample (50–100 µg of protein) was mixed with an equal volume of 2 × electrophoresis sample buffer and denaturation was achieved by incubating the mixture at 95 °C for 10 min. After gel electrophoresis, semi-dry electroblotting was used to transfer the proteins onto a nitrocellulose (PVDF) membrane. The membrane was first blocked by shaking in 5% bovine serum albumin (BSA) in Tris-buffered saline containing 0.05% Tween for 1 h. The membrane was then incubated with the primary antibody overnight at 4 °C followed by incubation with secondary antibodies (coupled to horseradish peroxidase). Signals were detected using enhanced chemi luminescence (ECL) reagent according to the manufacturer’s instructions.

### Histopathological evaluation of hepatic injury

Histopathological investigation was performed as previously descibed (Akool 2015).

### Immunohistochemical evaluation of hepatic expression of TLR4

Immunohistochemical staining was performed as previously described (Mounieb et al. 2017). Paraffin-embedded sections of 4 µm thickness were deparaffinised in xylene and rehydrated in graded ethanol solutions to distilled water. In order to block nonspecific immune responses, sections were then incubated with 5% bovine serum albumin in Tris buffered saline for 2 h. For immunostaining, sections were then incubated with the primary antibody TLR4 in a dilution of 1:100 at 4 °C overnight. After washing with TBS, sections were incubated with secondary antibody (goat anti-rabbit) for 1 h at room temperature. After washing, sections were incubated with diaminobenzidine (DAB) for 5 min at room temperature. The slides were washed then counterstained with hematoxylin. Positive immunoreactions were visualized under a light microscope.

### Statistical analysis

For statistical analysis of various groups, GraphPad prism 9.3.1 Demo (GraphPad software, San Diego, CA) was used. Results were analyzed using one-way ANOVA, followed by Tukey’s multiple comparisons test, and reported as mean ± SD. The level of significance was set at *P* < 0.05.

## Results

### LD NX reduced ALT and AST levels induced by Con A

**Fig. 1:**
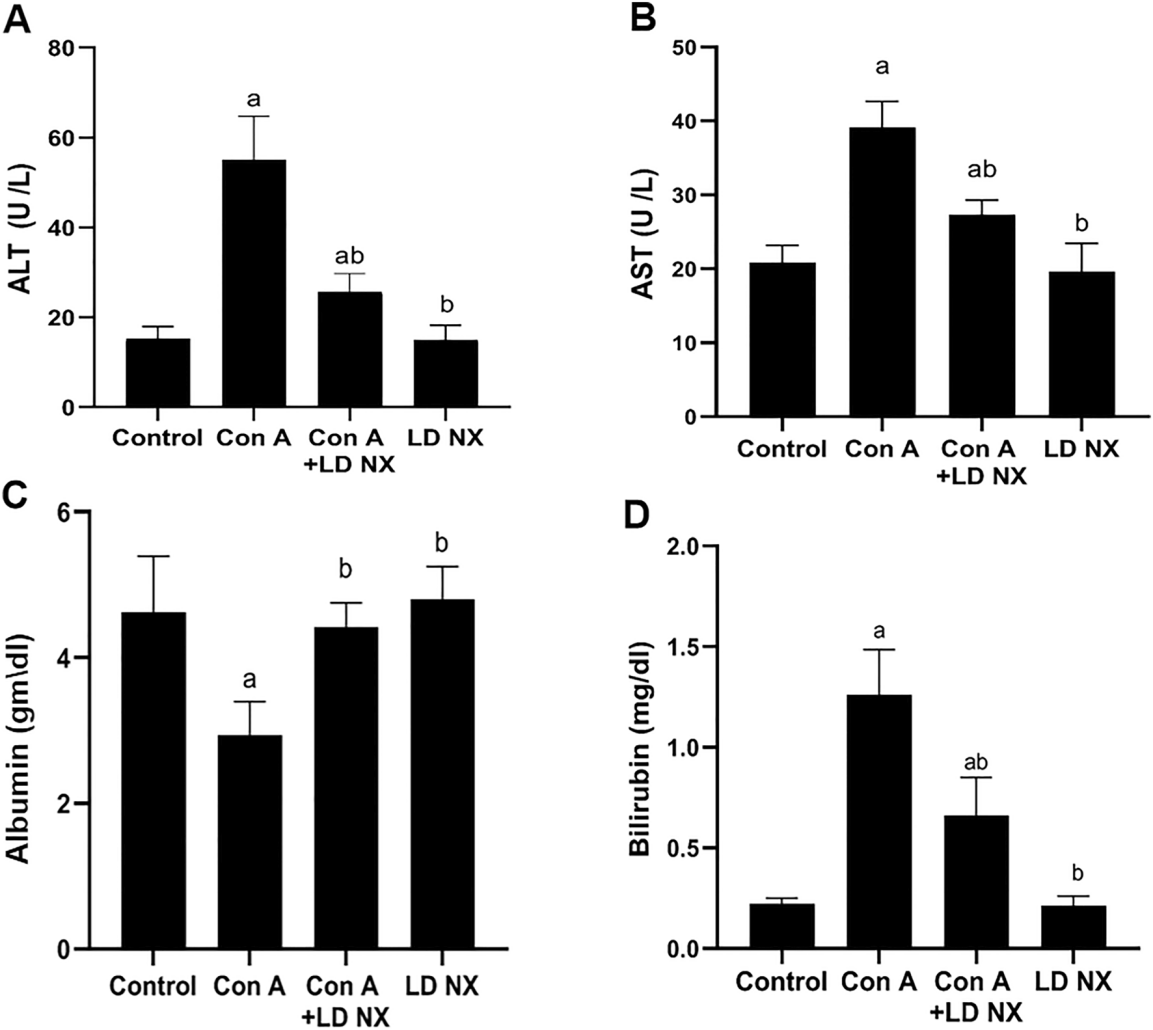
Effects of Con A and/or LD NX on serum ALT (**A**), AST (**B**), albumin (**C**), and bilirubin (**D**) levels in male Wistar albino rats. Data represent means ± S.D. (*n* = 6), ^a^Significantly different from control group at *p* < 0.001, ^b^Significantly different from Con A group at *p* < 0.001. *Con A* Concanavalin A, *LD NX* low dose naloxone, *ALT* alanine aminotransferase, *AST* aspartate aminotransferase

As shown in Fig. 1A and B, serum ALT and AST levels were significantly increased in Con A-treated rats as compared to control. However, serum ALT and AST levels were highly reduced in animals pretreated with LD NX before Con A injection as compared to animals treated with Con A alone. No significant changes were observed in animals treated with LD NX alone.

### LD NX up-regulates albumin production in Con A-treated animals

Treatment of animals with Con A significantly reduced albumin production compared with control group (Fig. 1C), indicating deterioration of hepatic function. However, LD NX pre-treatment significantly improved liver function as indicated by an increase in albumin level compared with Con A-alone treated group (Fig. 1C). Nothing changed in animals treated with LD NX alone.

### LD NX down-regulates bilirubin level induced by Con A

Administration of Con A significantly increased bilirubin level as compared to control group (Fig. 1D). On the other hand, pre-treatment of animals with LD NX before Con A injection significantly reduced bilirubin level as compared to Con A alone treated animals. No significant changes in bilirubin level were observed in LD NX alone-treated animals.

### LD NX attenuates Con A-induced lipid peroxidation

**Fig. 2:**
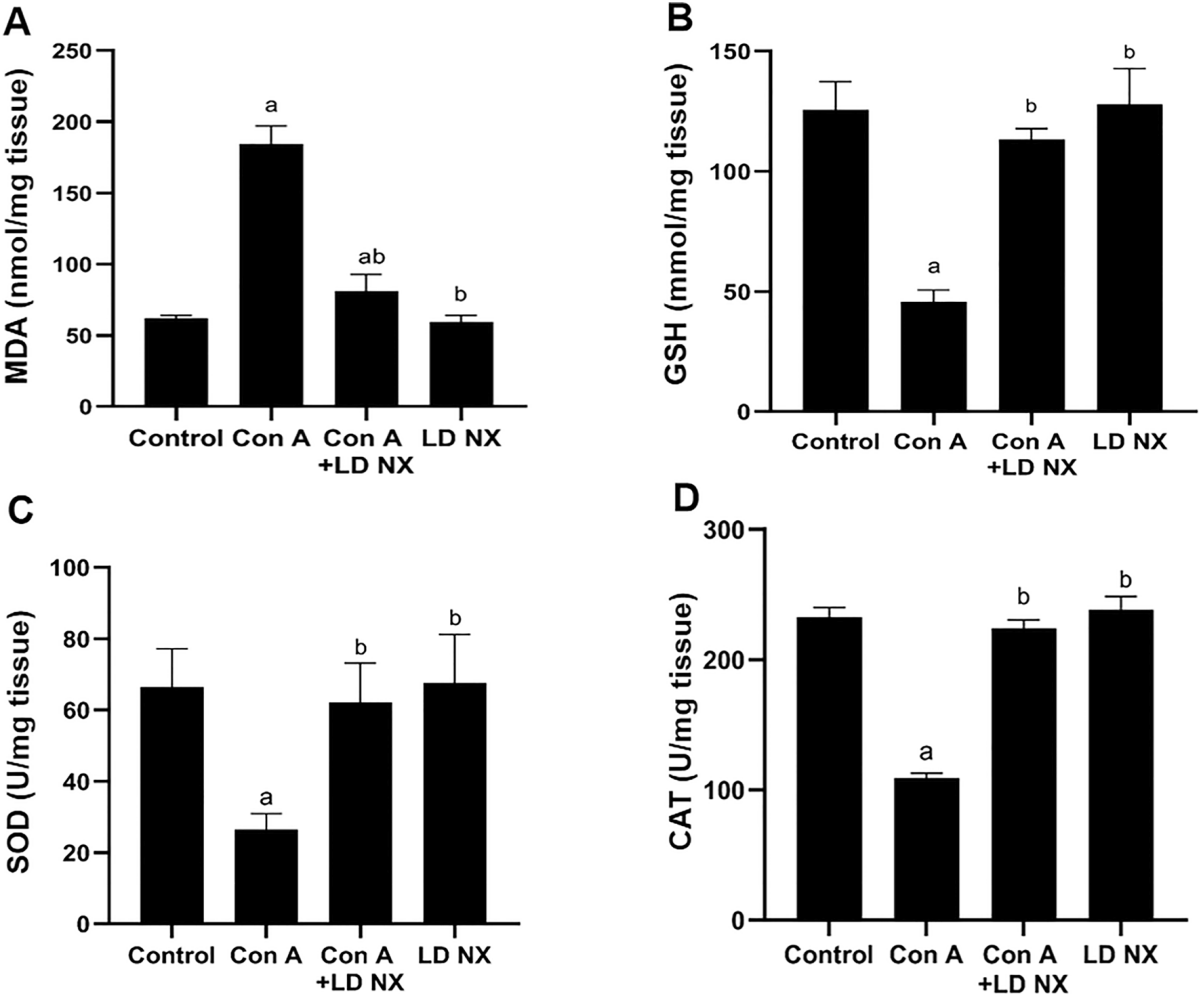
Effects of Con A and/or LD NX on the hepatic content of MDA (**A**), GSH (**B**) as well as SOD (**C**) and CAT (**D**) activities in male Wistar albino rats. Data represent means ± S.D. (*n* = 6), ^a^Significantly different from control group at *p* < 0.001, ^b^Significantly different from Con A group at *p* < 0.001. *Con A* Concanavalin A, *LD NX* low dose naloxone, *MDA* Malondialdehyde, *SOD* superoxide dismutase, *CAT* catalase, *GSH* Reduced glutathione

Treatment of animals with Con A significantly induced lipid peroxidation as indicated by an increase in the by-product of lipid peroxidation (MDA) (Fig. 2A), indicating the oxidative damage induced by Con A in the liver. However, pre-treatment of animals with LD NX significantly attenuated lipid peroxidation induced by Con A (Fig. 2A). The liver content of MDA in animals treated with LD NX alone was not changed.

### LD NX increases GSH content in animals treated with Con A

Treatment of rats with Con A significantly reduced GSH content in liver tissues (Fig. 2B). However, GSH content in animals pre-treated with LD NX before administration of Con A was significantly increased compared with Con A alone treated animals. No significant changes were detected in LD NX alone-treated rats.

### LD NX increases SOD and CAT activities in Con A-treated animals

As shown in Fig. 2, administration of Con A significantly attenuated SOD and CAT activities. On the other hand, SOD and CAT activities were significantly increased in animals pre-treated with LD NX before Con A administration as compared to Con A alone treated animals (Fig. 2C, D). The activities of SOD and CAT in LD NX alone treated animals were not changed.

### LD NX reduces the pro-inflammatory cytokines induced by Con A in the liver

**Fig. 3:**
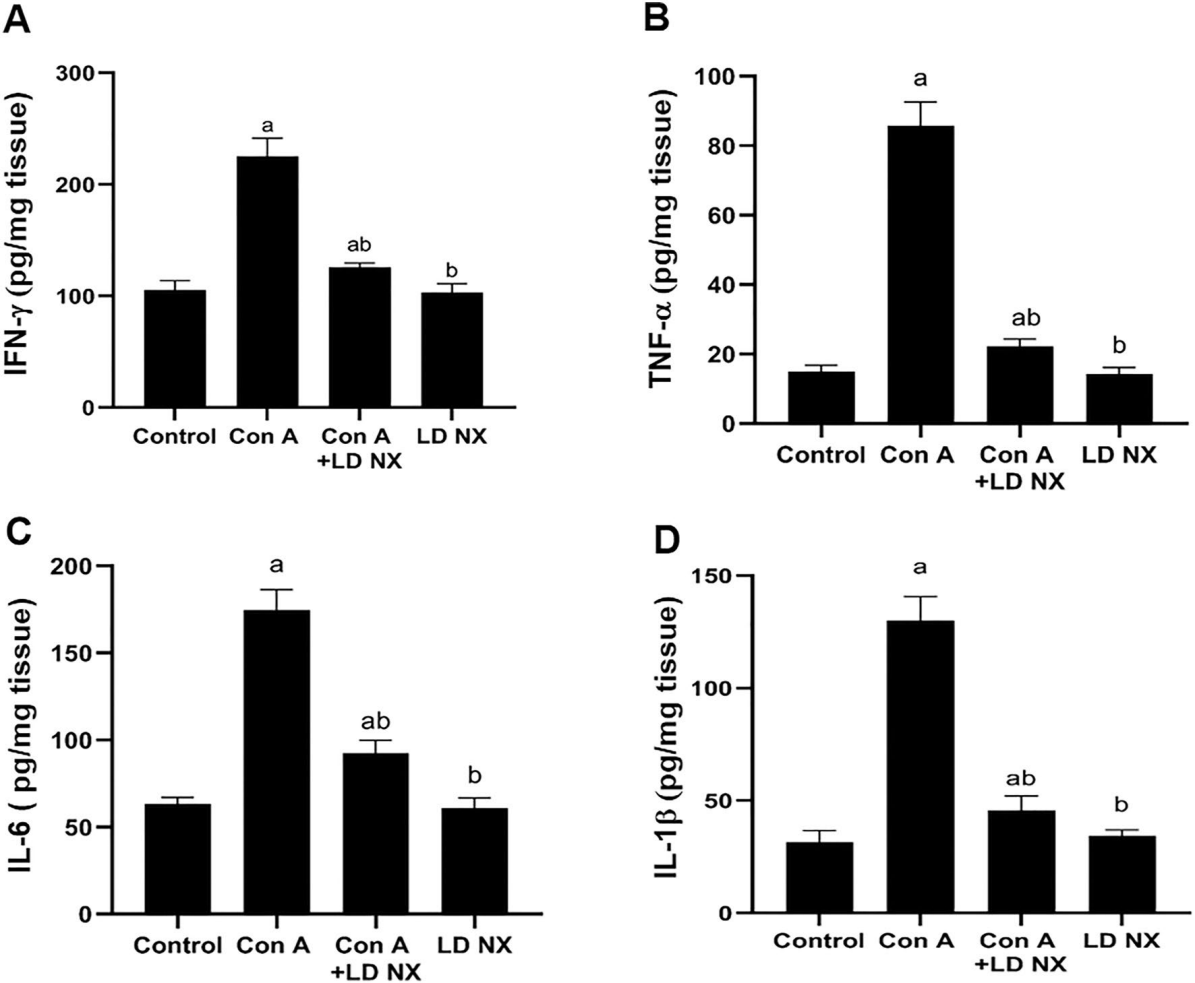
Effects of Con A and/or LD NX on liver content of inflammatory cytokines, IFN-γ (**A**), TNF-α (**B**), IL-6 (**C**), IL-1β (**D**). Data represent means ± S.D. (*n* = 6), ^a^Significantly different from normal control group at *p* < 0.001, ^b^Significantly different from con A group at *p* < 0.001. *Con A* Concanavalin A, *LD NX* low dose naloxone, *IFN-γ* Interferon-γ, *TNF-α* Tumor necrosis factor-α, *IL-6* Interleukin-6, *IL-1β* Interleukin-1β

The possible changes in the pro-inflammatory cytokines IFN-γ, TNF-α, IL-6 and IL-1β were assessed. It was observed that Con A administration significantly increased the hepatic levels of the pro-inflammatory cytokines IFN-γ, TNF-α, IL-6 and IL1β. However, pre-treatment of animals with LD NX before Con A injection significantly reduced IFN-γ, TNF-α, IL-6 and IL-1β levels as compared to animals treated with Con A alone (Fig. 3A–D, respectively). The levels of IFN-γ, TNF-α, IL-6 and IL1β were not changed in rats treated with LD NX alone.

### LD NX inhibits NF-κB expression induced by Con A

**Fig. 4:**
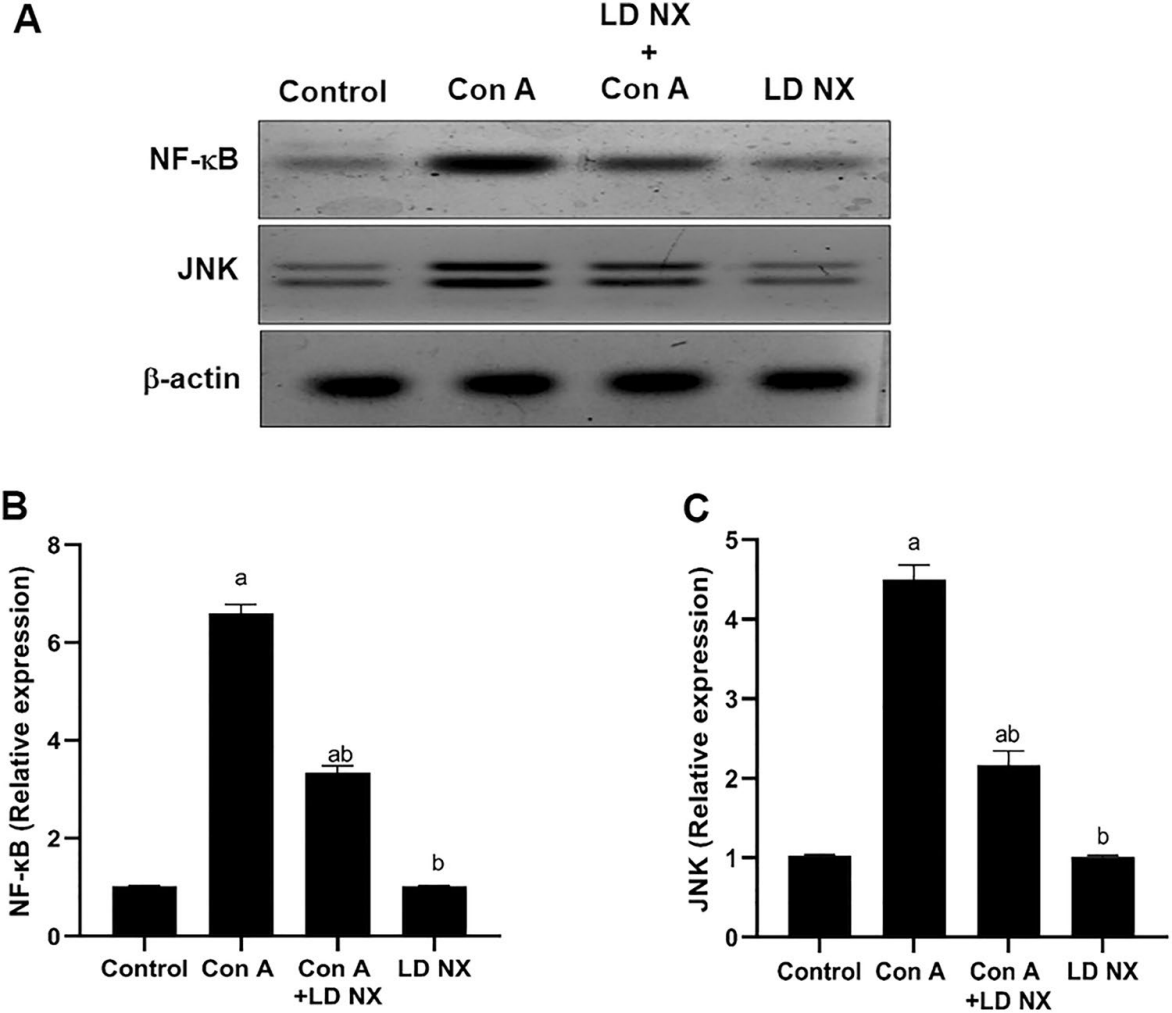
Total liver extracts from rats were subjected to Western blot analysis and probed with anti-NF-κB, anti-JNK and β-actin (**A**). The rats were given either vehicle (control) or Con A (12 mg/kg, i.v.) or LD NX (5 μg/kg/daily, i.p.) or Con A in conjunction with LD NX. B. A densitometric analysis of NF-κB in relation to the level of β-actin C. A densitometric analysis of JNK in relation to the level of β-actin. Data represent means ± S.D. (*n* = 3), ^a^Significantly different from normal control group at *p* < 0.001, ^b^Significantly different from con A group at *p* < 0.001. *Con A* Concanavalin A, *LD NX* low dose naloxone

As shown in Fig. 4, Con A administration significantly induced NF-κB expression. On the other hand, LD NX pre-treatment significantly reduced the expression of NF-κB as compared to Con A alone treated rats. The expression of NF-κB was not changed in animals treated with LD NX alone.

### LD NX attenuates JNK expression induced by Con A

Administration of Con A significantly induced JNK expression. However, pre-treatment of animals with LD NX before Con A injection significantly reduced the expression of JNK as compared to Con A alone treated rats (Fig. 4). The expression of JNK was not changed in LD NX alone-treated rats.

### LD NX attenuates TLR4 expression in rat liver

**Fig. 5:**
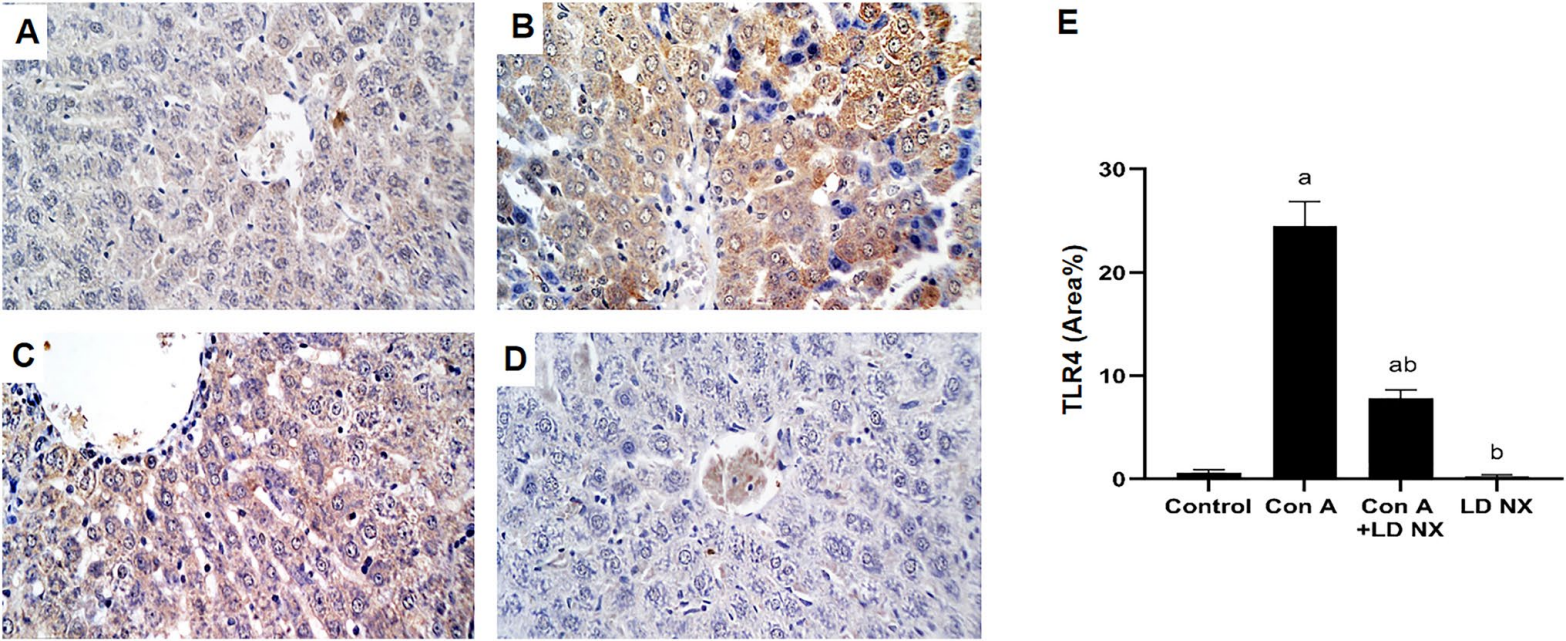
LD NX reduces the expression of TLR4. Immunohistochemical staining of TLR4 in liver tissues from rats treated with either vehicle (control) or Con A (12 mg/kg, i.v.) or LD NX (5 μg/kg/daily, i.p.) or Con A in conjunction with LD NX. **A** Liver of control group animals showing mild expression of TLR4 within the normal hepatocytes. **B** Liver of Con A group animals showing marked multifocal expression of TLR4 within hepatic tissues. **C** Liver of Con A + LD NX group animals showing moderate expression of TLR4 within the hepatic tissues. **D** Liver of LD NX group animals showing mild expression of TLR4 within the hepatic tissues. **E** Quantification of TLR4 area percentage. Data are presented as mean ± S.D. (*n* = 3), ^a^Significantly different from control group at *p* < 0.05, ^b^Significantly different from Con A group at* p* < 0.05. *LD NX* low dose naloxone, *Con A* Concanavalin A, *TLR4* toll-like receptor 4

The expression of TLR4 was significantly increased in Con A alone-treated animals. On the other hand, LD NX significantly attenuated the expression of TLR4 induced by Con A in rat liver as compared to Con A alone-treated animals (Fig. 5). No significant changes were observed in LD NX alone-treated animals.

### LD NX enhances Nrf2 and HO-1 expression in rat liver

**Fig. 6:**
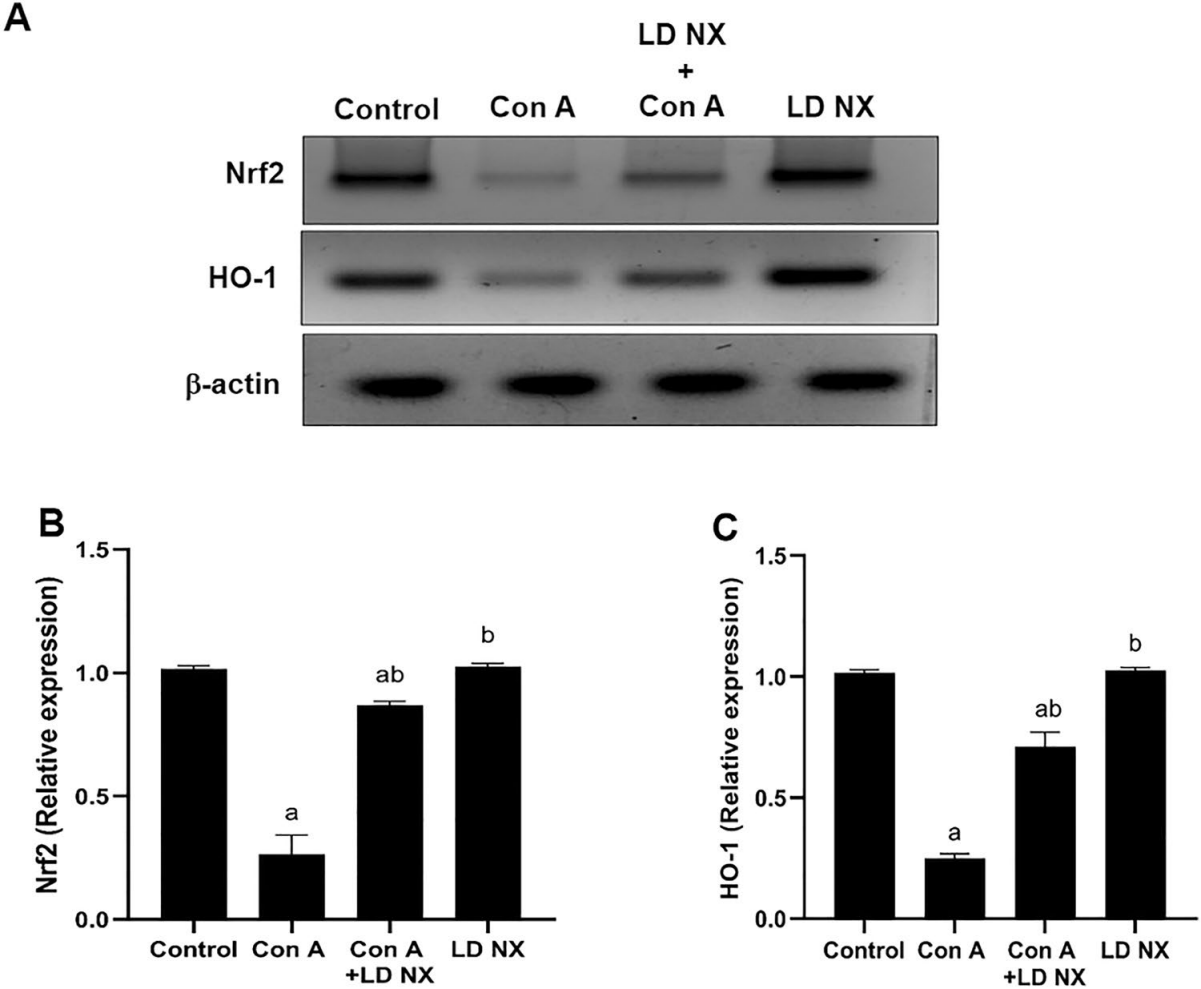
**A**Total liver extracts from rats were subjected to Western blot analysis and probed with anti-Nrf-2, anti-HO-1 and β-actin. The rats were given either vehicle (control) or Con A (12 mg/kg, i.v.) or LD NX (5 μg/kg/daily, i.p.) or Con A in conjunction with LD NX.** B** A densitometric analysis of Nrf-2 in relation to the level of β-actin C. A densitometric analysis of HO-1 in relation to the level of β-actin. Data represent means ± S.D. (*n* = 3), ^a^Significantly different from normal control group at *p* < 0.001, ^b^Significantly different from con A group at *p* < 0.001. *Con A* Concanavalin A, *LD NX* low dose naloxone

As shown in Fig. 6, injection of Con A attenuated Nrf2 expression in rat liver. However, pre-treatment of rats with LD NX before Con A administration increased the expression of Nrf2 as compared to Con A-alone treated group. As the downstream of the Nrf2 signaling pathway, HO-1 expression was also reduced in Con A group. On the other hand, HO-1 expression significantly increased in animals pre-treated with LD NX before Con A injection compared with Con A alone-treated animals.

### LD NX improves histopathological changes induced by Con A in rat liver

**Fig. 7:**
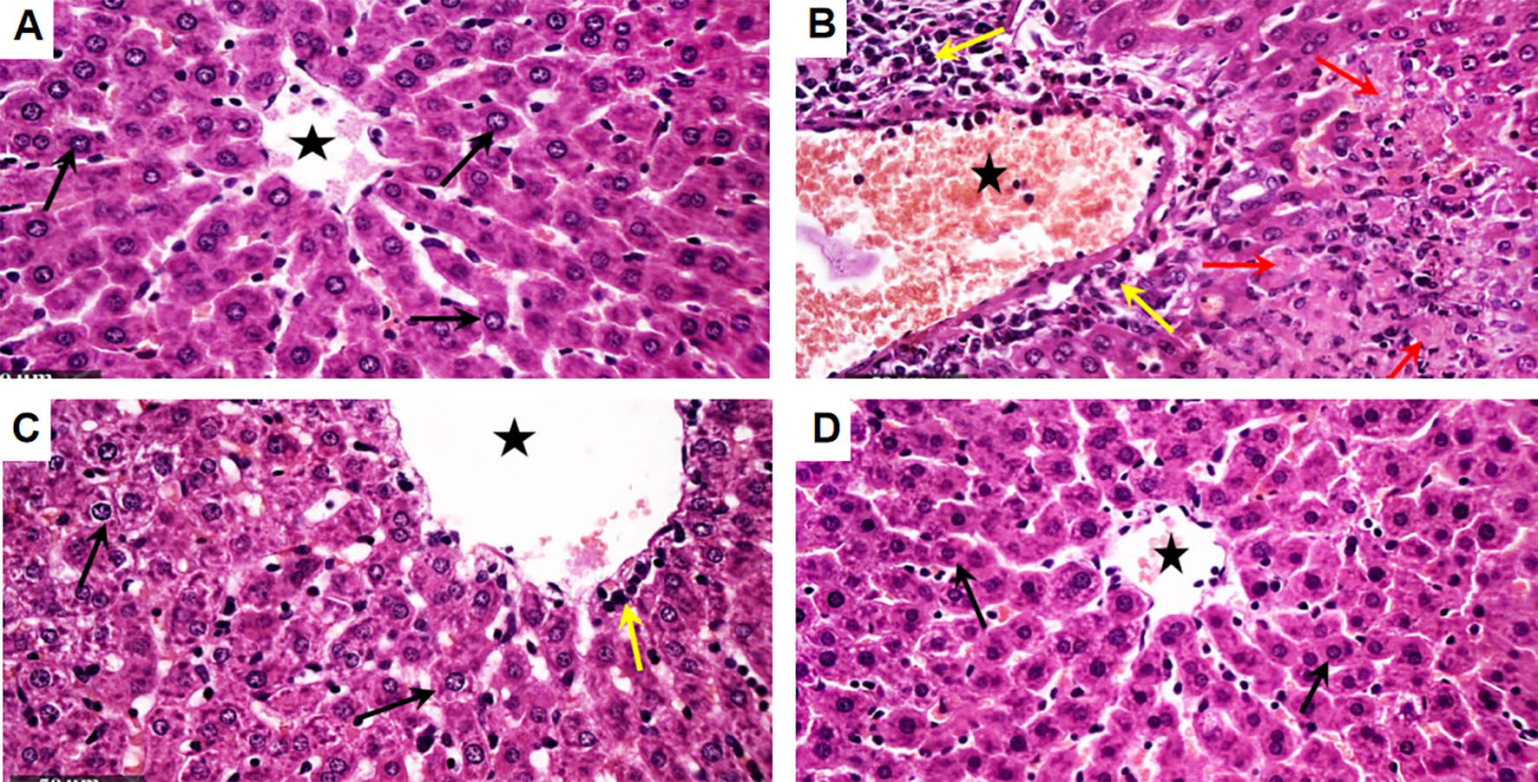
Photomicrographs of liver sections stained by hematoxylin and eosin (× 200). **A** Section taken from control group showing normal histological features of rat liver parenchyma with many apparent intact well organized hepatocytes with intact subcellular details (black arrow) and minimal sporadic records of degenerated hepatocytes, intact hepatic vasculatures (star) **B** Section taken from Con A-treated group showing multiple figures of focal hepatocellular necrosis (red arrow) all over most of hepatic lobules accompanied with marked dilatation of hepatic vasculatures (star) with sever perivascular mononuclear inflammatory cells infiltrates (yellow arrow). **C** Section taken from a rat treated with LD NX + Con A showing more organized histological features of hepatic parenchyma with abundant records of apparent intact hepatocytes (black arrow) with minimal records of degenerative and necrotic changes. Occasional persistent records of dilatation of hepatic blood vessels (star) with lesser extensive records of inflammatory cells were also observed (yellow arrow). **D** Sections taken from LD NX group showing apparent intact hepatic parenchyma resembling normal controls. *Con A* Concanavalin A, *LD NX* low dose naloxone

As demonstrated in Fig. 7, liver sections of control group demonstrated normal histological features of rat liver parenchyma with many apparent intact well organized hepatocytes with intact subcellular details and minimal sporadic records of degenerated hepatocytes, intact hepatic vasculatures, as well as hepatic sinusoids were shown without abnormal changes records (Fig. 7A). While those of Con A group showed multiple figures of focal hepatocellular necrosis all over most of hepatic lobules. Also, a marked dilatation of hepatic vasculatures were showed with sever perivascular mononuclear inflammatory cells infiltrates (Fig. 7B). Liver sections of Con A + LD NX group showed more organized histological features of hepatic parenchyma with abundant records of apparent intact hepatocytes with minimal records of degenerative and necrotic changes (Fig. 7C). Occasional persistent records of dilatation of hepatic blood vessels with lesser extensive records of inflammatory cells were also observed. The liver sections of rats treated with LD NX alone revealed apparent intact hepatic parenchyma resembling normal controls (Fig. 7D).

## Discussion

Naloxone is a non-selective opiate receptors antagonist (Van Dorp et al. 2007), that is mainly used in acute opioid overdose or intoxication (Brewer and Wong 2004). Recently, opiate antagonist has been shown to provide protection against a variety of inflammatory disorder such as Parkinson’s disease, Alzheimer’s disease, and multiple sclerosis when used off label but within a specific dosage window (Medina-Rodriguez et al. 2020; Tewari et al. 2021). It has been shown that the opioid receptors antagonist naltrexone can be used for drug withdrawal and prevention of relapse at the label dosage of 50 mg/day. Also, naltrexone has been used to treat chronic pain syndrome and autoimmune diseases at a dose of 5 mg/day (Li et al. 2018). High doses of naloxone induce the expected hyperalgesic effects, while low doses cause antinociceptive effects (Tsuruoka et al. 1997). Intrathecal injection of ultra-low dose naloxone has been shown to improve the antinociceptive effect of morphine by suppressing microglial activation, pro-inflammatory cytokines TNF-α, IL-1β and IL-6 in the spinal cord of pertussis toxin-treated rats (Lin et al. 2010). Low dose naltrexone is thought to exert its anti-in-flammatory effects through non-opioid antagonist mechanisms. It has been demonstrated that naltrexone has also the ability to inhibit non-opioid receptors such as TLR-4 in macrophages and microglia (Cant et al. 2017; Li et al. 2018). Here, in this study, we tried to investigate the potential hepatoprotective effects of LD NX against AIH induced by Con A.

In the current study, serum hepatic function tests revealed a significant elevation in serum levels of ALT, AST, and bilirubin in animals treated with Con A. Also, a significant reduction in serum level of albumin and a clear histopathological alterations were observed in Con A alone-treated rats, indicating a decline in liver function as a result of the hepatotoxic effect of Con A. These data are in agreement with previous studies (El- Kashef and Abdelrahman 2020; Mounieb et al. 2017). Interestingly, these findings were significantly improved in animals pre-treated with LD NX, indicating that LD NX has the ability to attenuate liver injury induced by Con A.

Several studies have shown ROS as a significant element in Con A-induced acute liver damage (Shirin et al. 2010; Zhuang et al. 2016). The present study demonstrates that Con A administration induces hepatic oxidative stress as indicated by a significant elevation in hepatic MDA and significant depletion in hepatic GSH as well as a clear reduction in SOD and CAT activities in rats treated with a single injection of Con A. These results are in the line with a previous study (Zhuang et al. 2016). Our results demonstrate that pre-treatment with LD NX before Con A injection reveal a high protection against Con A-induced oxidative stress as indicated by a clear reduction in hepatic MDA and restoration of the hepatic antioxidants GSH, SOD and CAT. These findings corroborated prior study demonstrated that opioids antagonist are powerful antioxidants (Ebrahimkhani et al. 2006). It has been shown that naloxone has the ability to reduce the formation of reactive oxygen species by inhibiting microglia activation (Younger et al. 2014). The Nrf2/HO-1 pathway has been shown to play a significant role in the regulation of several cytoprotective genes including antioxidant ones that protects against oxidative damage (Khodir et al. 2017). In this study, pre-treatment with LD NX boosted the cellular antioxidant defense mechanism as indicated by an increase in Nrf2 and HO-1 expression.

TLR4 is known to be involved in drug-induced liver damage, which can be resolved by blocking it (Ishida et al. 2021). Several investigators found that TLR4 was key in the pathophysiology of Con A-induced liver injury (Sahin et al. 2013). It plays a crucial role in the inflammatory signaling responses to various stimuli, leading to the transcription of a variety of inflammatory genes in NF-κB-dependent pathway (Gargiulo et al. 2015). Recently, it has been demonstrated that Chinese propolis protects vein endothelial cells from inflammation by interfering with MAPK/NF-κB signaling pathway (Xuan et al. 2019). Moreover, it has been reported that activation of TLR4/NF-κB signaling pathway plays an important role in the initiation of innate immune response and subsequent release of inflammatory molecules (Gargiulo et al. 2015). It has been demonstrated that Con A has the ability to activate T cells to secrete a variety of hepatotoxic cytokines, TNF-α, IFN-γ, IL-1β and IL-6 (Mounieb et al. 2017; Tiers et al.^1998^). Previously, it has been reported that TNF-α and ROS may both function as positive feedback signals to activate NF-κB (Shirin et al. 2010). Also, it has been demonstrated that Nrf2 regulates the expression of NF-κB. Notably, NF-κB can also regulate Nrf-2 expression, implying a complex interdependence or bidirectional interaction between these pathways (Que et al. 2023). In our study, the expression of TLR4 was elevated in the Con A group, however, LD NX pre-treatment before Con A injection dramatically reversed this overexpression. Furthermore, pre-treatment with LD NX significantly attenuated the release of the inflammatory molecules, TNF-α, IFN-γ, IL-1β and IL-6. Moreover, NF-κB expression was highly reduced in animals pre-treated with LD NX before Con A injection. Recently, it has been shown that curcumin has anti-inflammatory properties via blocking NF-κB and JNK signaling pathways (Ruan et al. 2022). Furthermore, it has been demonstrated that JNK plays an important role in the development of hepatitis (Das et al. 2009). In our study, JNK expression was significantly reduced in rats pre-treated with LD NX before administration of Con A. Considering that TLR4 plays a key role in controlling inflammation, we hypothesized that LD NX exerts its hepatoprotective impact in this model, at least in part, via inhibition of TLR4 expression. Supporting our data, Hutchinson and his colleagues stated that naloxone and naltrexone have the ability to block TLR4 signaling (Hutchinson et al. 2008). Moreover, TLR4 antagonism has contributed to various effects of naloxone treatment that include inflammation that were described prior to its identification as a TLR4 antagonist. In animal models, naloxone, for example, demonstrated an anti-inflammatory impact against sepsis (Medina-Rodriguez et al. 2020). LD NX is thought to exert its anti-inflammatory effects through non-opioid antagonist pathways (Li et al. 2018). According to our findings, LD NX considerably activated the Nrf2/HO-1 pathway, which may, in turn, suppress the activation of TLR4/NF-κB pathways, reducing the release of the inflammatory cytokines. So, its hepatoprotective effect may account for its anti-inflammatory impact and antioxidant effect.

Finally, our findings demonstrate for the first time that pre-treatment of LD NX before Con A administration significantly attenuated all hepatic damage markers and improved liver function indicating that LD NX has the ability to protect rats from Con A-induced hepatitis via modulation of TLR4/NF‑κB and Nrf2/HO‑1 pathways.

## Conclusion

The present study demonstrates for the first time the hepatoprotective effect of LD NX against Con A-induced autoimmune hepatitis which may be related to its ability to suppress inflammatory cytokines secretion and interfere with ROS generation via modulation of TLR4/NF-κB, Nrf2/HO-1 and JNK signaling pathways. Our findings provide new insights into the use of LD NX for AIH management. Clinical trials are needed to investigate the potential curative effects of LD NX on liver injury.

## Data Availability

Data that support the study's findings are presented in the publication or supplemental data.
